# Supplementary material for: Ru-N-C Hybrid Nanocomposite for Ammonia Dehydrogenation: Influence of N-doping on Catalytic Activity
Source: Materials (Basel). 2015 Jun 10;8(6):3442–55. doi: 10.3390/ma8063442 (PMC5455761; doi:10.3390/ma8063442)
Supplement: Supplementary file 1 [file materials-08-03442-s001.pdf]

Supplementary Materials

Table S1. The Ru, N, and C contents of Ru-N-C and Ru-C.

Catalysts	Ru (%) [a]	C (%) [b]	N (%) [b]
Ru-C	0.86	98.4	0
Ru-N-C	0.97	84.8	14.2

Notes: [a]: determined by ICP; [b]: measured by SEM-EDX.

Table S2. The NH_3 conversions at the GHSV of 7448 $mL \cdot g^{-1} \cdot h^{-1}$ as a function of temperature.

Temperature (°C)	C-N	Ru-C	Ru-N-C
475	0	0	24
500	0	20	56
550	0	68	95

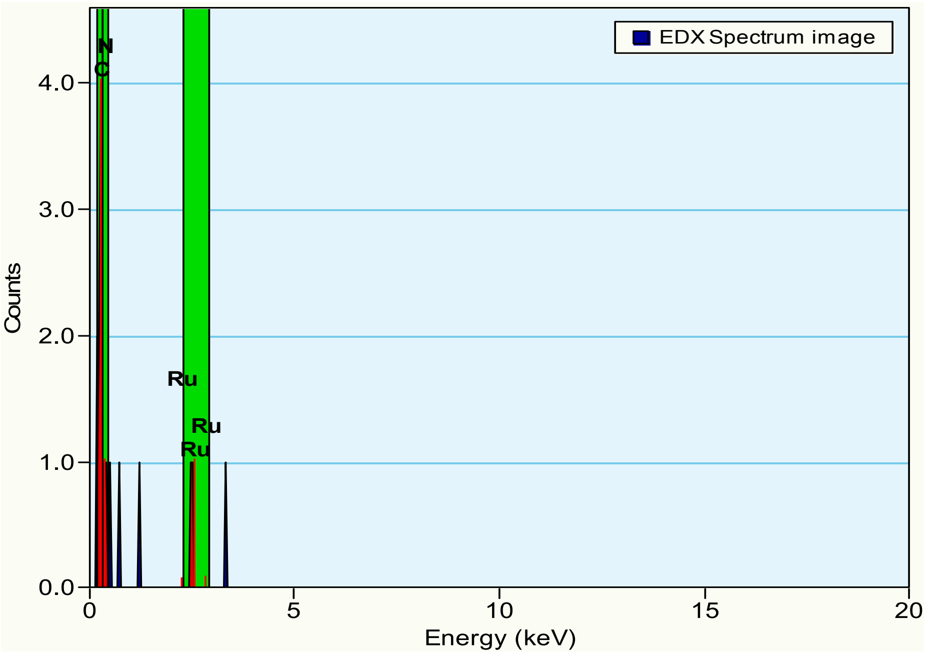

Figure S1. The EDX spectrum of Ru-N-C.

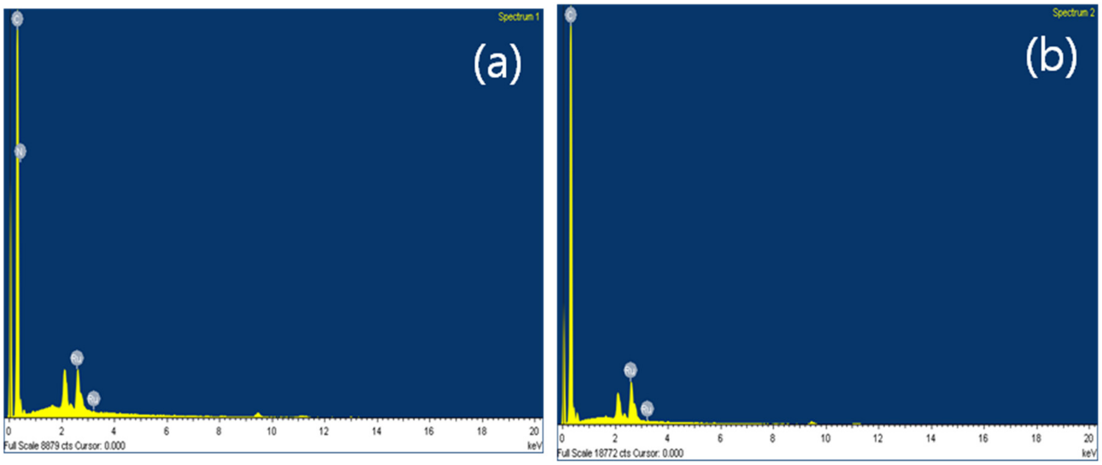

Figure S2. EDS spectra: (**a**) Ru-N-C and (**b**) Ru-C.

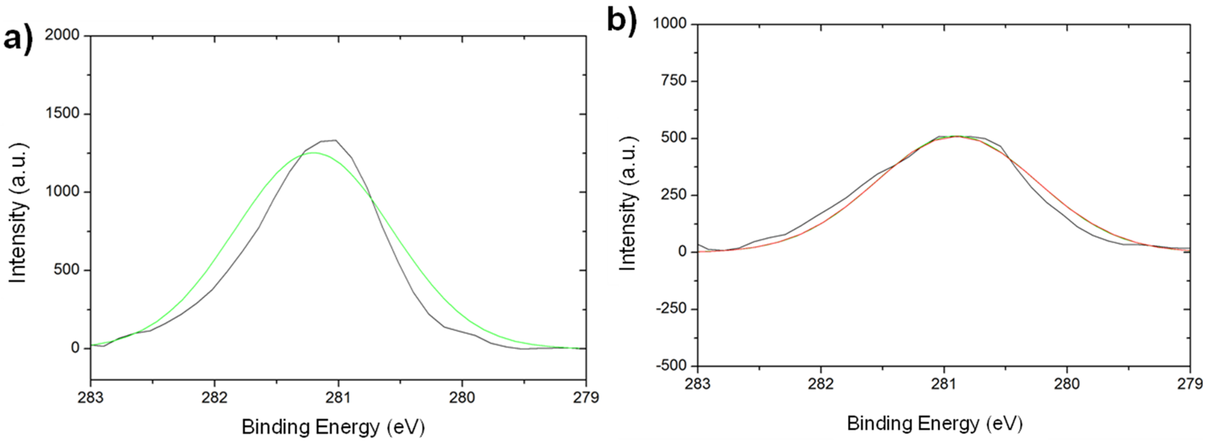

Figure S3. XPS Ru 3d Ru-C (**a**); and Ru-N-C (**b**) respectively.

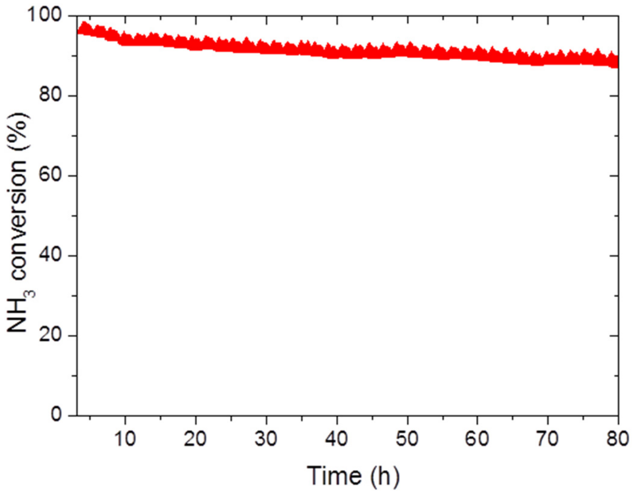

Figure S4. Long term stability for NH_3 dehydrogenation over Ru-N-C at 550 °C with a GHSV of 7448 mL·g^{-1}·h^{-1}.

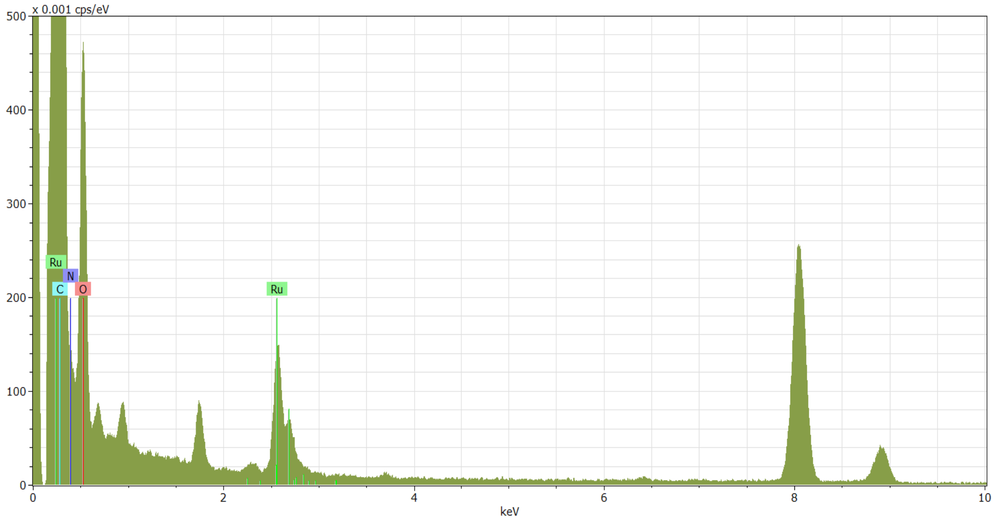

Figure S5. The EDX spectrum of the spent Ru-C catalyst after the long-term stability test (80 h).
